# *Artemisia abrotanum* L. (Southern Wormwood)—History, Current Knowledge on the Chemistry, Biological Activity, Traditional Use and Possible New Pharmaceutical and Cosmetological Applications

**DOI:** 10.3390/molecules26092503

**Published:** 2021-04-25

**Authors:** Halina Ekiert, Ewa Knut, Joanna Świątkowska, Paweł Klin, Agnieszka Rzepiela, Michał Tomczyk, Agnieszka Szopa

**Affiliations:** 1 Chair and Department of Pharmaceutical Botany, Medical College, Jagiellonian University, ul. Medyczna 9, 30-688 Kraków, Poland; ewa.knut@gmail.com (E.K.); asiek.pajor@student.uj.edu.pl (J.Ś.); 2 Family Medicine Clinic, Medizinisches Versorgungszentrum (MVZ) Burgbernheim GmbH, Gruene Baumgasse 2, 91593 Burgbernheim, Germany; bag-burgbernheim@gmx.de; 3 Museum of Pharmacy, Medical College, Jagiellonian University, ul. Floriańska 25, 31-019 Kraków, Poland; agnieszka.rzepiela@uj.edu.pl; 4 Department of Pharmacognosy, Faculty of Pharmacy, Medical University of Białystok, ul. Mickiewicza 2a, 15-230 Białystok, Poland; michal.tomczyk@umb.edu.pl

**Keywords:** *Artemisia abrotanum*, southern wormwood, traditional medicinal use, chemical composition, biological activity, potential medicinal value, position in cosmetology, biotechnological studies

## Abstract

*Artemisia abrotanum* L. (southern wormwood) is a plant species with an important position in the history of European and Asian medicine. It is a species famous as a medicinal plant in Central Asia, Asia Minor, and in South-East and Central Europe. The raw materials obtained from this species are *Abrotani herba* and *Abrotani folium*. In the traditional European medicine, they have been used successfully most of all in liver and biliary tract diseases, in parasitic diseases in children and as antipyretic medication. In the official European medicine, this plant species is recommended by the French Pharmacopoeia for use in homeopathy. In many European countries, it is used traditionally in allopathy. The latest studies on the biological activity of extracts from the aboveground parts of the plant and/or the leaves, and/or the essential oil have provided evidence of other possible applications related to their antibacterial, antifungal, antioxidant, anticancer, and antiallergic properties. The latest studies have also focused on the repellent activity of the essential oil of this species and the possibility to use it in the prevention of diseases in which insects are the vectors. The main substances obtained from the plant that are responsible for this activity are: the essential oil, coumarins, phenolic acids, and flavonoids. Some of the latest investigations emphasize the large differences in the composition of the essential oil, determined by the geographical (climatic) origin of the plant. *A. abrotanum* is recommended by the European Cosmetic Ingredients Database (CosIng) as a source of valuable cosmetic ingredients. Additionally, the leaves of this species possess a well-established position in the food industry. This plant species is also the object of biotechnological studies.

## 1. Introduction

The awarding of the 2015 Nobel Prize in Medicine for the discovery of artemisinin [1,2], a sesquiterpenoid lactone, in *Artemisia annua* (annual mugwort) and proving its effectiveness in the treatment of malaria sparked a huge interest in the world of science in the chemistry and biological activity of other *Artemisia* species [3,4,5,6,7]. One of the better-known medicinal species of this genus in Europe and Asia is *Artemisia abrotanum* L. (Asteraceae). This species occurs in the south-eastern part of Europe and in the countries of Central and North-Western Europe. This species also has its natural habitats in Asia Minor, Central Asia, and on the Arabian Peninsula [8]. The herb of this species—*Abrotani herba*— occupies an important position in the traditional medicine of European countries [8,9,10,11]. It is recommended most often for the treatment of diseases of the liver and the biliary tract. It is also used as an effective anthelmintic in children, and as an antipyretic. Recent professional studies have proven previously unknown biological activities of extracts from the herb and/or the leaves, and/or the distilled-off essential oil, namely antibacterial, antifungal, antioxidant, anticancer, and antiallergic properties [8,12]. Apart from the essential oil, the coumarins, phenolic acids and flavonoids present in the chemical composition of the plant are also responsible for the abovementioned biological effects. This species is also traditionally an object of interest of the food industry [13]. A recent development was its approval by the European CosIng (Cosmetic Ingredients) database for use in the production of cosmetics and its growing role as a cosmetic plant [14]. The aim of this article is to present the current state of knowledge regarding the position of *A. abrotanum* in the history of medicine, its importance in modern traditional medicine, the chemical composition, new directions of biological activity and the resulting possible new therapeutic applications and prospects for use in cosmetology. The aim was also presentation of biotechnological studies of this species and its position in the food industry.

## 2. Position in the History of European and Asian Medicine

*A. abrotanum* is one of those medicinal herbs that can be found already in the first herbals of our civilization. Descriptions of it were included in both Dioscorides and Pliny the Elder [15,16]. Noteworthy is the origin of the name “*abrotanum*”, which, according to Theophrastus of Eresos, may refer to a certain constitution of the body, namely to very thin and frail people (αβρος (abrós) in Greek means ‘slim, delicate’) [17]. This reference is significant because it corresponds exactly to the constitution of patients who were originally recommended to use this herb, namely those suffering from chronic respiratory ailments. Of the lung diseases, the most difficult to treat over the millennia had been tuberculosis, leading to persistent wasting of the body, but chronic bronchitis or asthma may have clinically manifested themselves in this way as well. In holistic Traditional Chinese Medicine (TCM), this condition is called “Lung-Qi weakening, with particular weakening of the Yin of the lungs”, and is the dominant indication for TCM treatment with this herb to this day [18]. Apart from bronchopulmonary ailments, *A. abrotanum* was also used in a number of other diseases, and its effects were widely known. In the Arab world, Avicenna, citing Galen among others, recommended its use in the absence of appetite, ulcers, and skin diseases, for hair growth, and as an amulet against evil spirits [19]. The Slavs considered *A. abrotanum* to be a cult “church herb”, used, for example, for making wreaths, hence its Polish name “boże drzewko” (“God’s little tree”) [20]. In the Middle Ages, applications from antiquity were adopted, and in Germanic areas in the 10th century, the spectrum of treatments with *Artemisia abrotanum* included broadly understood difficulty in breathing, pain from the liver, spleen, bladder and uterus, anthelmintic treatment, wound healing, to assist the stomach, intensify diuresis, and deter evil spirits [21]. A hundred years later, Hildegard of Bingen insisted on using this herb in compresses for leg pain [22]. In the Renaissance era, due to the warming and drying properties of *A. abrotanum*, it was recommended for use in ailments caused by cold and humidity, which mainly concerned the female genital tract and the functional circuits of the liver and spleen, according to the holistic nomenclature. This view was shared by the “Fathers of Botany”—Otto Brunfels [23], Hieronymus Bock [24], and Leonhart Fuchs [25], and half a century later also by their worthy successor—Jacobus Tabernaemontanus [26]. At the apex of the development of holistic thought in Western medicine, the following secondary properties were attributed to *A. abrotanum*: “*dispersing*, *diluting*, *(surface) opening and cleansing effects”* [27]. One of the most important Polish renaissance herbals—“Zielnik” by Simon Syrenius, also mentions the plant’s drying and warming effects as well as its effectiveness as a remedy against “venoms and poisons” (after Dioscorides and Pliny), repelling “venomous animals”, and in diseases of the lungs and the digestive system, and in women’s diseases [28]. *A. abrotanum* was also used as an antipyretic, against internal parasites and externally in the treatment of scalp and hair diseases, and as an anti-inflammatory medication in eye diseases and ulcers of various origins. The plant was used to prepare an oil with analgesic and diuretic properties, wine stimulating the appetite and effective against jaundice, and vodka for respiratory ailments [28]. In 19th-century folk medicine, the herb *A. abrotanum* was known as a medicine for skin diseases (added to baths), for throat and ear ailments, for sprains (in the form of compresses), and for women’s and childhood diseases [29].

## 3. General Information on the Species

*A. abrotanum* has many synonymous Latin names, including *Abrotanum alpestre* Jord., *A. ambiguum* Jord. & Fourr., *A. brachylobium* Jord. & Fourr., *A. congestum* Jord. & Fourr., *A. incanescens* Jord. & Fourr., *A. mas* Garsault, *A. pauciflorum* Jord. & Fourr., *A. pedunculare* Jord. & Fourr., *A. platylobum* Jord. & Fourr., *A. pulverulentum* Jord. & Fourr., *A. rhodanicum* Jord. & Fourr., *A. suave* Jord. & Fourr., *A. virgatum* Jord. & Fourr., *A. viridulum* Jord. & Fourr., *A. xerophilum* Jord. & Fourr., *Artemisia abrotanifolium* Salisb., *A. altissima* Ehrh., *A. altissima* Ehrh. ex DC. *A. anethifolia* Fisch., *A. anethifolia* Fisch. ex DC. [30,31,32]. The species is also known by numerous common names, for example: lad’s love, lemonwood, old man, slovenwood, southern wormwood, and southernwood (in English), and nayqatamisa (ajmara), abrotone, armoise aurone, armoise citronnelle, aurone mâle, aurone, citronelle aurone, citronnelle (in French). In German, there are only two names—Eberraute and Stabwurz [31,32,33,34,35,36]. In Polish, the species is customarily called “boże drzewko” (literally “God’s little tree”, a counterpart of the English name “Our Lord’s wood”) [33]. The pharmaceutical raw material is *Artemisiae abrotani herba*—the herb of the *A. abrotanum* plant—the flowering tips of its shoots. The dry herb has a grey-green colour, a slightly bitter taste and a citrusy, spicy aroma [33,37]. In traditional medicine, the leaves of the plant—*Artemisiae abrotani folium*, also constitute the raw material [11]. *A. abrotanum* is a semi-shrub reaching a height of 0.7–1.5 m [38]. The shoots of this species grow upright and have soft twigs. Young twigs are blue-green and older ones are brown. The stem is covered with secretory hairs [39]. The stems are highly branched with dense foliage. The grey-green leaves have numerous covering hairs on the upper side; the underside of the leaves is smooth. The leaves growing in the lower part of the stem are doubly pinnate and have ensiform sections, while in the upper parts they take on a singly pinnate, tripartite, and also ensiform shape. The whole plant has a strong distinctive lemon-like aroma [5,33,37,40,41].

The tiny yellow tubular flowers are gathered in spherical or ovoid-spherical hanging heads that form panicles. In Central Europe, flowering of the plant begins in August and lasts until September. Due to the climatic conditions, the plant in this region does not bear fruit. The fruits are small oblong achenes [5,33,37,40,41]. Cariological studies of the plant have shown that its specimens are most often diploid (2n = 2x = 18), less often tetraploid (2n = 4x = 36) [38]. *A. abrotanum* is a species native to Central Asia—in particular Armenia, Iran and Russia, and also to Asia Minor—Turkey and Europe, including Albania and Croatia [36]. The species also occurs in Central and North-Western Europe [42].

*A. abrotanum* likes warm, sunny, and humus-rich sites, with well-drained, not too moist soils [33,43]. In Central and North-Western Europe, the species reproduces only vegetatively by division or from hardwood and softwood cuttings. In other parts of the world, if the species grows in a warm climate, it reproduces by seed. The first harvest takes place in the second year after planting. During the flowering period, the tips of the shoots are collected, which are then dried in shaded and airy natural drying enclosures. Larger batches of the raw material are dried in drying rooms with the temperature increased to 35 °C. It is also advisable to trim annually the lower, lignified parts of the plant. This treatment improves the branching and ‘bouncing-back’ of the herb, and increases the yield [33,43]. After drying, the woody parts are discarded and the non-lignified herb is placed in airtight packages and stored in the dark [39,41,43].

## 4. Chemical Composition

Analyses of the compounds present in *A. abrotanum* have focused primarily on the essential oil present in the herb of the species [9,11,40]. The composition and concentration of *A. abrotanum* essential oil are variable and dependent on the cultivation site, among other things [9]. The dominant fraction of the oil are compounds with a monoterpenoid structure. The remaining fractions are compounds of sesquiterpenoid, diterpenoid, triterpenoid, or spiroterpenoid structures, and phenylpropanoid derivatives (Table 1).

In 2020, Lithuanian researchers determined the composition of the essential oil obtained from the herb of *A. abrotanum* plants growing in Lithuania, which were at various stages of plant development. The compound that was present in the highest amounts was piperitone. The remaining substances isolated in large amounts were: 1,4-cineole, lavandulyl butanoate, aromadendrene, and isogermacrene D. The essential oil content in the herb of the plant was the highest at the stage of flower-bud setting (38.48%), and the lowest at the stage of leaf growth and development (20.38%) [9].

Egyptian researchers conducted a study in 2020 evaluating an influence of trace elements on productivity of *A. abrotanum*. The main components isolated from essential oil of *A. abrotanum* aerial parts were: 2-hydroxy-1,8-cineole, β-eudesmol, and camphor. In the experiment the scientists treated *A. abrotanum* with foliar spray containing iron (Fe), magnesium (Mg) or manganese (Mn). The foliar application of Fe (3 g/L), Mg (8 g/L) or Mn (300 mg/L) significantly increased values of growth, yield, major constituents of essential oil and chemical composition of the plant in comparison with control [46]. 

**Figure 1:**
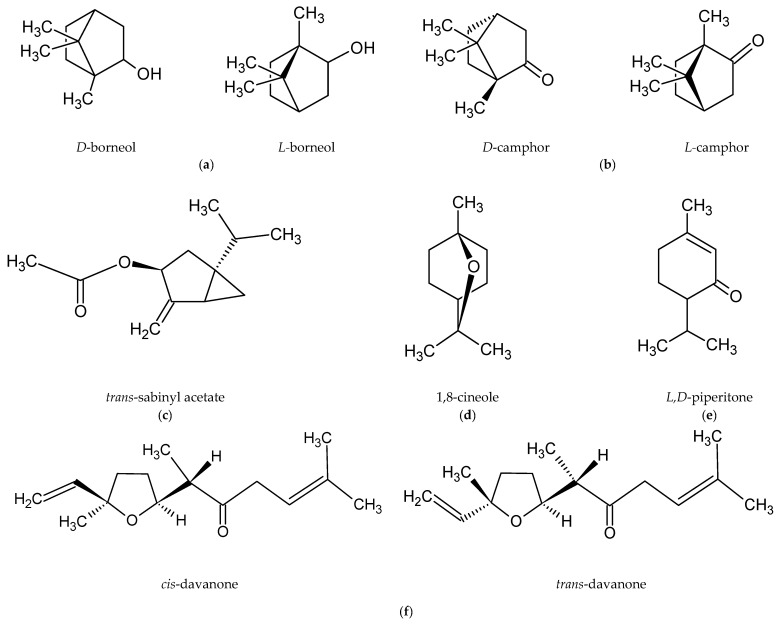
Chemical structure of the dominant compounds in *A. abrotanum* essential oil: (**a**) borneol; (**b**) camphor; (**c**) *trans*-sabinyl acetate; (**d**) 1,8-cineole; (**e**) piperitone; (**f**) davanone.

Romanian scientists have reported that the main component of the essential oil of *A. abrotanum* herb was davanone and its derivatives. The researchers also isolated, in small amounts, 1-terpineol, *trans*-piperitol, and estragole [47]. In contrast, the dominant compound in the essential oil of plants harvested in Cuba was *trans*-sabinyl acetate [44], and in the essential oil from plants harvested in Iraq borneol [48]. The main components in plants harvested in Crimea were 1,8-cineole and camphor [49]. The chemical structures of the most abundant compounds in *A. abrotanum* essential oil are shown in Figure 1.

**Figure 2:**
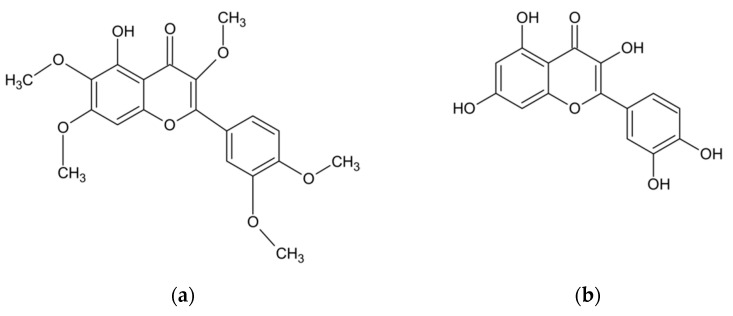

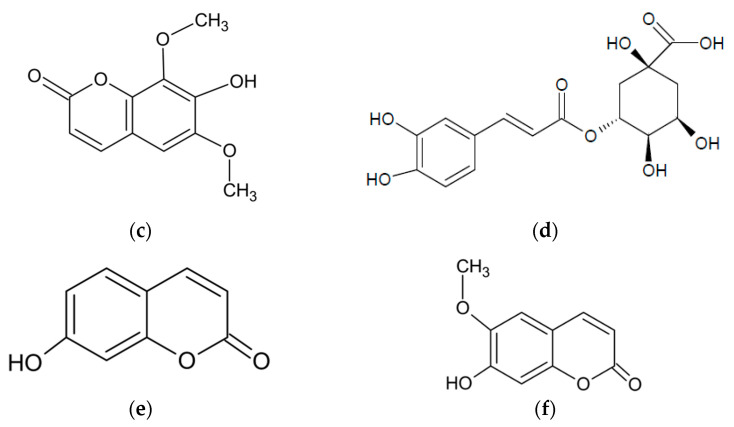
Chemical structure of the most frequent compounds isolated from *A. abrotanum*: artemisetin (**a**); quercetin (**b**); isofraxidine (**c**); chlorogenic acid (**d**); umbelliferone (**e**), scopoletin (**f**).

In addition to the essential oil, the herb of *A. abrotanum* also contains compounds with phenolic structures. The research conducted at JSS University in Mysore (India) has documented the presence of phenolic compounds in the amount of 9.35 mg/g dry weight in terms of gallic acid, and the presence of flavonoids in the amount of 11.0 mg/g dry weight in terms of rutoside [34]. Another study has proven the presence of phenolic acids. This group of compounds, present in the leaves of *A. abrotanum* plants growing in Saudi Arabia, was dominated by rosmarinic, chlorogenic, caffeic and isochlorogenic acids [40]. The structures of the compounds are shown in Figure 2. Other studies, in turn, identified the flavonoids present in the plant, including apigenin, hyperoside, quercetin, quercitrin, kaempferol, luteolin, myricetin, rutoside, and also lactone - artemisinin [33,37,40]. Among other compounds isolated from the herb of *A. abrotanum* were also coumarins, including isofraxidine, umbelliferone, scopoletin, herniarin, and esculetin [33,37,47,50,51]. Moreover, the herb has been found to contain the next sesquiterpenoid lactone—santonin [50], an alkaloid—abrotine [33,37], tannins, organic acids [44], sterols [52], and resin [33,37,53,54]. The chemical composition of the *A. abrotanum* plant is shown in Table 2.

## 5. Application in Traditional European and Non-European Medicine

In European traditional medicine, *Abrotani herba* is used in diseases of the liver, such as atony, contractile states of the bile ducts and stagnation of or insufficient bile secretion. Herbal infusions are used to improve digestion, increase appetite, and also as an astringent in diarrhea. It is also recommended to use infusions as an aid in cases of anorexia, flatulence, and hypoacidity [14,33,37,39]. Extracts from the plant have also been used as a remedy for frostbite, lymphadenitis, and in the treatment of epilepsy [37,39,43]. The species has been used in the treatment of malaria [54].The leaves have been used to improve liver function, stimulate menstruation, and relax smooth muscles. Other indications have included cancer prophylaxis and therapy, and men have used the plant as a hair growth agent [55]. The plant is also used as an anthelmintic agent in the treatment of oxyurosis and ascariasis in children [33]. In Bosnia and Herzegovina, the species has also been used in the treatment of jaundice [58], and in Turkey in the treatment of fever [59]. In traditional Indian medicine, Siddha, *A. abrotanum* is used as a repellent to repel insects and parasites [60].

## 6. Application in Modern Phytotherapy and Position in Official European Medicine

The herb *A. abrotanum* can be found primarily in homeopathic medicines. The French Pharmacopoeia states that their production is based on a 65% mother tincture, prepared from fresh herb of *A. abrotanum*. The tincture should contain at least 0.1% m/m of *ortho-*dihydroxycinnamic acid derivatives, expressed in terms of chlorogenic acid [61,62]. These preparations are indicated for the treatment of inflammation of the colon, rosacea, frostbite, and inflammation of the lymph nodes and mucous membranes [37,43]. Homeopathic medicines from *A. abrotanum* are also indicated for people suffering from depression and anxiety [63]. The European Medicines Agency (EMA) has also authorized the use of homeopathic preparations from *A. abrotanum* in farm animals used for food production [11].

*Artemisiae abrotani herba* and *Artemisiae abrotani folium* do not have the status of pharmacopoeial raw materials in European countries. The species and the raw materials obtained from it are, however, successfully used in official European allopathic medicine. A good example is the research conducted at Havelhoehe Research Institute and Charite’ University Medical Centre in Berlin, and the University of Witten/Herdecke in Herdecke (Germany). For a period of two years (2004–2005), it was recorded the prescribing by primary care doctors and specialist doctors of preparations containing plant extracts from plants of the family Asteraceae. Over that time, preparations with *A. abrotanum* extracts were recommended to 202 patients. The preparations included both homeopathic remedies and herbal mixtures as well as medicines containing only *A. abrotanum* extract. The indications for the use of these medications were usually diagnosed non-infectious inflammations of the intestines or of the large intestine [45,61]. 

In official European medicine similar as in traditional medicine the most often the whole plant extracts are used [11]. They contain the mixture of flavonoids, coumarins, phenolic acids and essential oils. These groups of metabolites with polyphenolic structures are important plant components with among others antioxidant and antitumor activities. For documented antibacterial and antifungal properties the components of essential oil are responsible. Synergic action might be created between these groups of polyphenols. In the phytotherapy as the galenic formulation the infusions and tinctures from *A. abrotanum* herb are recommended. Moreover, this raw material is endorsed for use in choleretic and gastric mixtures, as well as for strengthening and soothing baths. *A. abrotanum* is also the subject of patents in the field of pharmacy in Europe [43,64]. The mechanisms of action of *A. abrotanum* extracts and/or essential oil are summarized in Table 3.

## 7. New Directions of Biological Activity of Extracts from the Herb and/or Leaves, and/or Essential Oil Confirmed by Scientific Research

### 7.1. Antibacterial and Antifungal Activity

The studies of the antibacterial and antifungal activities of ethanolic extracts from the herb of *A. abrotanum* were performed by Suresh et al. Using the disc diffusion method and the serial dilution method, the effect of the extracts on the bacteria: *Bacillus subtilis*, *B. stearothermophilus*, *Micrococcus luteus*, *Klebsiella pneumoniae*, *Pseudomonas cepacia*, *Salmonella typhi*, and the fungi: *Candida albicans*, *Saccharomyces cerevisiae*, *Trichosporon beigelii*, was tested. The control treatment was performed with 1 mg/mL penicillin for the bacteria and 500 μL/mL amphotericin B for the fungi. The extract proved to be effective against all the pathogens except *B. subtillis*. The maximum zone of growth inhibition among the bacteria was obtained for *P. cepacia* (28.6 mm) after using the *A. abrotanum* ethanolic extract at 30 mg/mL. The inhibition zone for the control was 31.4 mm. Among the fungi, significant activity was demonstrated with the use of 30 mg/mL extract for *T. beigelii* (17 mm) and *S. cerevisiae* (17 mm), compared with the control samples for which the zones of growth inhibition were 21 mm and 26 mm, respectively. It was also shown that the antimicrobial effect of the extract depended on its concentration [65].

Researchers from Romania have examined the activity of the essential oil obtained from the herb of *A. abrotanum* against a strain of *Candida albicans*. The disc diffusion method was also adopted as the test method. The measured zone of growth inhibition was 23.5 mm for the oil obtained by steam distillation and 21.5 mm for the oil obtained by extraction with dichloromethane. The growth inhibition zone for nystatin as the control was 20.5 mm. The results of the study indicate that *A. abrotanum* exhibits stronger antifungal activity than nystatin, which is a medication commonly used in the treatment of fungal infections [47].

Iranian researchers have also investigated the activity of the essential oil of *A. abrotanum* against pathogenic microorganisms. They tested the effect of the oil on the following bacteria: *Staphylococcus aureus*, *Bacillus subtillis*, *Salmonella typhi*, *Escherichia coli*, and the fungus *Candida albicans*. For this purpose, they used the method of diffusion in agar wells. The use of 15, 25 or 40 μL of the essential oil was shown to inhibit the growth of *S. aureus*, while the use of 10, 15, 25 or 40 μL of the essential oil inhibited the growth of *E. coli* and *C. albicans*. The authors of the study indicate that the antimicrobial effect of *A. abrotanum* can be attributed to the compounds contained in the plant, such as borneol, cymene, camphor, terpineol, 1,8-cineole, and aromadendrene [63].

Another study determined the activity of the essential oil isolated from the herb of *A. abrotanum* against the bacteria *Escherichia coli*, *Pseudomonas aeruginosa*, *Staphylococcus aureus* and *Proteus vulgaris*, and against the fungi *Candida albicans*, *Aspergillus flavus*, and *Fusarium oxysporum*. The experiment was carried out using the disk diffusion method. With 25 μL of the essential oil, the zone of growth inhibition was 19.67 mm for *E. coli*, 10.33 mm for *P. aeruginosa*, 16.89 mm for *S. aureus*, and 18.89 mm for *P. vulgaris*. For the control with 10 μL/mL gentamicin, the zones of growth inhibition were 11 mm, 25 mm, 25 mm, and 23 mm, respectively. Among the tested fungi, the essential oil showed activity only against *A. flavus* (the growth inhibition zone was 11.89 mm after using 25 μL of a 75% solution of the essential oil diluted with Tween 40) [66].

In the another study, the action of the methanolic extract from the *A. abrotanum* plant and of the individual components of the extract were tested against the bacteria: *Listeria monocytogenes*, *Staphylococcus aureus*, *Escherichia coli*, *Bacillus cereus*, *Pseudomonas aeruginosa* and *Micrococcus flavus*, and against the fungi: *Penicillium ochrochloron*, *P. funiculosum*, *Candida albicans*, *Aspergillus ochraceus*, *A. niger* and *A. flavus* using the microdilution method. The extract showed good antibacterial properties and moderate antifungal properties against the pathogens tested. The action of quercitrin and isochlorogenic acid against the bacteria was shown to be as strong as that of streptomycin, which served as a positive control [55].

Ukrainian researchers have conducted a study evaluating antimicrobial activity of *Artemisia* L. herb extracts, including *A. abrotanum* extract. Therefore, the team has prepared ethanol extracts of *A. abrotanum*, *A. vulgaris and A. absinthium* with three concentration of the solvent: 40%, 70% and 90%. Micromethod of diffusion in agar has been employed in antimicrobial activity assessment. Moderate inhibition of the growth of bacteria: *Streptococcus pyogenes*, *Streptococcus agalactiae*, *Streptococcus gordonii*, *Enterococcus faecalis*, *Escherichia coli*, *Citrobacter freundii*, *Pseudomonas aeruginosa*; methicilin suseptible: *Staphylococcus aureus and Staphylococcus epidermis*; methicillin resistant: *Staphylococcus aureus* and *Staphylococcus haemolyticus* and macrolides resistant: *Propionibacterium acnes* strains under influence of *A. abrotanum* herb ethanolic extract has been noted. The strongest bacteriostatic activity of the 70% ethanol extract has been displayed against *Propionibacterium acnes* MLS (8.71 mm. = inhibition zone) in comparison with the control (70% ethanol). The results also showed a decrement of the growth of *Candida albicans* and *Candida tropicalis* colonies as well as a significant inhibition of *Aspergillus niger* spore germination (13.32 mm.). Moreover, the study has also evaluated a synergistic action of *A. abrotanum* ethanol herb extract with erythromycin against *Staphylococcus aureus* with efflux mechanism of MLS-resistance. Only extract with 90% ethanol displayed a synergistic action with erythromycin. The results demonstrated inhibition zone 6.83 mm. with 1.95 µg/mL concentration of erythromycin and 7.48 mm. with 31.25 µg/mL of erythromycin. Of note, erythromycin alone didn’t exhibit any action against *Staphylococcus aureus* with efflux mechanism of MLS-resistance so did medium without erythromycin [12].

### 7.2. Antioxidant Effect

The antioxidant potential of an ethanolic extract from *A. abrotanum* herb was tested using the DPPH method. The results of the study showed moderate antioxidant activity of the plant extract. The IC_50_ value for the extract was 284.5 µg/mL, compared with the ascorbic acid control, for which the IC_50_ was 17.34 µg/mL [40].

Under another study the antioxidant activity of the essential oil isolated from the herb of *A. abrotanum* was tested. For this purpose, they determined the percentage inhibition of peroxidation of egg yolk lipids using the thiobarbituric acid method (TBARs). The reducing potential was investigated using the potassium hexacyanoferrate(III) method. In the TBARs test, 82.34% inhibition of lipid peroxidation was recorded after application of 1000 μL of the essential oil. The reducing potential of 100 μL of the oil was 135.97 μg of butylated hydroxyanisole equivalent [66].

The latest study conducted in 2020 has also confirmed the antioxidant activity of *A. abrotanum*. The effects of the methanolic extract from the leaves of *A. abrotanum* and of the individual components of the extract were determined using the β-carotene bleaching (BCB) method, the ability to reduce Fe(III) ions (FRAP test) and DPPH. The IC_50_ values recorded in the β-carotene bleaching test and the DPPH test were 35.4 μg/mL and 27.1 μg/mL, respectively, whereas for the butylated hydroxytoluene (BHT) control the IC_50_ was 2.7 μg/mL and 3.3 μg/mL, respectively. In the FRAP test, the IC_50_ was 39.1 μg/mL, whereas for the trolox control the IC_50_ was 3.2 μg/mL. The best results were obtained for rosmarinic acid (IC_50_ in the bleaching test was 3.1 μg/mL, 2.7 μg/mL in the DPPH test, and 3.5 μg/mL in the FRAP test) [55].

### 7.3. Antitumour Effect

The antitumour activity of the essential oil obtained from the herb of *A. abrotanum* was tested against the rhabdomyosarcoma (RD) cell line. The growth or inhibition of cancer cell proliferation was measured using the dye 3-(4,5-dimethylthiazol-2-yl)-2,5-diphenyltetrazolium bromide (MTT), which, when influenced by mitochondrial dehydrogenase present in living cells, changes colour from orange to dark blue. The viability of RD cells after the application of the essential oil at concentrations of 25, 50 and 100 μg/mL was 29.679%, 20.833%, and 20.256%, respectively. The negative control with DMSO and the positive control with methotrexate showed 32.179% and 18.205% survival of RD cells, respectively. The results of the study prove that the essential oil of *A. abrotanum*, especially in concentrations of 50 and 100 μg/mL, has an antitumour effect, probably due to the presence of compounds such as: borneol, cymene, camphor, terpineol, 1,8-cineole, and aromadendrene [63].

Under another study an ethanolic extract from the leaves of *A. abrotanum* and its individual components show antiproliferative activity against T-cell lymphoblastic leukemia cell lines (Jurkat line), breast adenocarcinoma (MCF-7 line), cervical adenocarcinoma (HeLa line), colorectal adenocarcinoma (HT-29 line), and embryonic human kidney cell lines (HEK-293 line). The MTT-mediated assay was used to determine the change in cell viability. The antiproliferative effect of the extract against all the lines, except for the HEK-293 line, was proven. Chlorogenic acid and isochlorogenic acid showed the greatest activity against these lines [55].

### 7.4. Allergy Symptom-Alleviating Effect 

Swedish researchers have assessed the effect of a nasal spray containing the essential oil and flavonoids derived from *A. abrotanum* on the course of allergic rhinitis. The essential oil contained large amounts of 1,8-cineole, davanone and linalool, with centaureidine dimethylether, casticin and quercetin dominant among the flavonoids. The preparation was administered to 12 patients with known allergic rhinitis and/or bronchial obstruction, or with allergic rhinitis with coexisting allergic conjunctivitis or with exercise-induced asthma. The preparation was applied immediately after noticing the characteristic allergic symptoms of the nose. The assessment of the preparation’s effects was based on a questionnaire filled in by patients who subjectively assessed the symptoms. Five minutes after application of the spray, the patients reported alleviation of nasal symptoms similar to the use of an antihistamine medication or chromoglycan. The soothing effect lasted up to several hours. Seven patients with symptoms of allergic conjunctivitis also reported improvement in ocular symptoms, and three patients with obstructive bronchial disease reported improvement in bronchial symptoms. The results of the study indicate that a nasal spray with a mixture of essential oils and flavonoids present in *A. abrotanum* can be effective in the treatment of allergic rhinitis and in the treatment of accompanying symptoms [56].

### 7.5. Insect-Repelling Action

The toluene extract from the herb of *A. abrotanum* were tested whether they could be used as a repellent. The effect of the plant extract was tested against *Ixodes ricinus* (common tick, vector of e.g., Lyme disease and tick-borne encephalitis) and *Aedes aegypti* (Egyptian mosquito, vector of e.g., Dengue virus) [50].

The experiment with *Ixodes ricinus* consisted in placing in a cage with ticks tissue papers soaked with the toluene extract from the *A. abrotanum* herb or with individual components of this extract. The control sample was tissue soaked only with ethanol. Records were kept of the tendency of ticks to avoid surfaces with the applied extract [50].

The experiment with *Aedes aegypti* was carried out in the open field and in a laboratory. In the laboratory, female mosquitoes were kept in a mesh enclosure and fasted for 2–3 days; then the tester placed in it a hand with the previously applied extract of *A. abrotanum* suspended in ethanol or with individual components of this extract. The other hand of the tester, without the extract applied, served as the control. The number of mosquitoes that landed on the hands was counted over a specified time period. In the field experiment, the tester similarly exposed a hand with the extract or its ingredients applied to it [50].

After 4 and 8 h from the time of applying the ethanolic suspension of the toluene extract from the herb *A. abrotanum*, the recorded repellency rates were, respectively, 69.1% and 56.8% against *Ixodes ricinus*, and 100% and 86.7% against *Aedes aegypti*. For the control sample with DEET (*N*,*N*-dietylo-m-toluamid), the repellency rates after 4 and 8 h were, respectively, 83.4% and 94.4% against *Ixodes ricinus*, and 100% and 95.8% against *Aedes aegypti*. The components that showed the highest percentage of repellency against *Ixodes ricinus* were camphor, coumarin and thujyl alcohol, and against *Aedes aegypti* coumarin, chlorogenic acid and caffeic acid [50].

### 7.6. Action against Animal Parasites

The antiparasitic activity of an ethanolic extract from the leaves of *A. abrotanum* has been investigated in an animal model (mouse). Rodents infected with *Hymenolepis nana* (dwarf tapeworm) or with rodent pinworms (*Syphacia obvelata* and *Aspiculuris tetraptera*) were given the ethanolic extract from *A. abrotanum* leaves, dissolved in water, for five consecutive days. The administration of the extract was shown to significantly reduce the egg count of all three parasites in the faeces of the mice. On the seventh day of therapy, no eggs were detected in the faeces. The research results indicate that the traditional use of *A. abrotanum* extracts as an anti-parasitic agent has been justified [67].

### 7.7. Antimalarial Activity

The potential antimalarial properties of silver nanoparticles from two *Artemisia* species: *A. abrotanum* and *A. arborescens* were studied. In the experiment, the hemocompatibilty of the nanoparticles using a microplate reader to measure the absorbance of hemoglobin release in supernatant were evaluated. The test was carried out on parasitized (*P. falciparum)* red blood cells (pRBCs). The cells were incubated for 24 and 48 h at 37 and 41 °C and at concentrations of nanoparticles: 0.6 µg/mL; 1.25 µg/mL; 2.5 µg/mL; 5 µg/mL and 7.5 µg/mL. Hemolysis assay revealed a better hemo-biocompatibility of *A. abrotanum* silver nanoparticles (*A. abrotanum*—AgNPs) than *A. arborescens* silver nanoparticles (*A. arborescens*—AgNPs). Moreover, hemolytic activity increased in dose dependent manner. To evaluate parasite growth inhibition the team exposed pRBCs to the influence of different nanoparticles in concentrations ranging from 0.6 to 7.5 µg/mL for 24 and 48 h. Significant inhibition of the growth of the parasite in the presence of *A. arborescens*—AgNPs has been observed.

In the next in vitro test evaluating an influence of the nanoparticles on maturation and death of *P. falciparum* parasite the scientists used non hemolytic concentration (2.5 µg/mL). Different mechanism of action of the two *Artemisia* species was noted. *A. abrotanum*-AgNPs exhibited antiplasmodial activity leading to parasite death in comparison with the control after 24 and 48 h of treatment. Contrariwise, *A. arborescens*-AgNPs antiplasmodial effect was associated with the parasite maturation stage blockage from trophozoite to ring stage. Futhermore, Avitabile et. al. measured IC_50_ of different silver nanoparticles. The results demonstrated that *A. abrotanum*-AgNPs displayed better antiplasmodial effect considering 50%, 90% and 99% inhibition concentrations [68].

The properties described above are summarized in Table 3.

## 8. Application in Cosmetology and in the Food Industry

*A. abrotanum*, like other *Artemisia* species, is also used in the production of cosmetics.The European CosIng database advises that *A. abrotanum* herb, leaf and stem extracts can be used as a protective, moisturizing, and caring agent for use on the skin [14]. Such extracts can be found in face and hand creams, serums, body lotions, scrubs, milks, and pedicure masks. Products containing *A. abrotanum* are offered by Polish companies such as *Organic life*, *Perfecta*, German: *Dr Hauschka*, English: *Bulldog Natural*, *Lush*, American: *Jack Black*, *Physicians Formula*, *Aveeno*, and French: *Decléor* [14]. Interestingly, according to a pilot study from 2021 *A. abrotanum* leaf extract offers promising results as an ingredient of nail gel used in patients with nail plate surface abnormalities [69]. Moreover, because of antibacterial activity against macrolides resistant *Propionibacterium acnes* strain, *A. abrotanum* extract has a potential as an active compound in cosmetics for acne-prone skin [12].

The leaves of *A. abrotanum*, due to their pleasant aroma, are used to flavour meats, salads, and cottage cheese. As flavourings, they are sometimes added to confectionery as well as to alcoholic beverages such as vermouths and liqueurs [13,33,41]. The herb of *A. abrotanum* can be found as an ingredient in teas [41].

## 9. Safety of Use

At Havelhoehe Research Institute and Charite’ University Medical Centre in Berlin, and the University of Witten/Herdecke in Herdecke (Germany), adverse effects associated with the use of preparations based on plants from the Asteraceae family had been documented for two years. Of the 236 patients taking homeopathic remedies, herbal mixtures, or single-ingredient preparations from *A. abrotanum* extracts, only two were found to produce side effects. The reported ailments, after taking a preparation composed of extracts of *A. abrotanum* and *Matricaria recutita*, included stomach pain and allergy. However, no serious side effects were reported [61].

## 10. Biotechnological Research

A review of the scientific literature has shown that *A. abrotanum* is a species that so far has sporadically been the object of research in the field of plant biotechnology. A protocol for the micro-propagation of this species has been only developed. Young leaves of the plant were sterilized and placed in the MS (Murashige and Skoog) medium supplemented with 4.44 µM 6-benzyladenine (BA) and 0.54 or 0.81 µM naphthyl-1-acetic acid (NAA). Abundant callus growth and shoot formation were achieved. With the addition of 4.44 μM BA and 0.54 μM of NAA, 3.61 shoots per explant were obtained (31 shoots in total), and with the addition of 4.44 μM BA and 0.81 μM NAA, 4.05 shoots per explant were obtained (total of 38 shoots). The obtained shoots were rooted using the MS medium without the growth regulators or supplemented with 0.49 µM indole acetic acid (IAA) or 0.54 µM NAA. Afterwards, the plants were transplanted into soil-filled pots [70].

## 11. Conclusions

*Artemisia abrotanum* is a species with an important position in the history of European, Middle-Eastern and Asian medicine (including especially Traditional Chinese Medicine (TCM)). Currently, it also has an important position in traditional European and Asian medicine (especially in the Middle East). The French Pharmacopoeia recommends the plant for use in official homeopathic medicine. In many European and Middle Eastern countries, this species is traditionally used in allopathic medicine. Recent phytochemical studies of this species have proven the variability of the chemical composition of its essential oil. Pharmacological studies, in turn, have provided evidence of new, previously unknown directions of biological activity of extracts from the herb and/or leaves, and/or essential oil of the plant—antimicrobial, antioxidant, anticancer, and antiallergic effects. Some studies also indicate the possibility of using the plant in the prevention of diseases spread by insects, due to the repellent properties of the essential oil. This creates prospects for the medicinal use of the plant to an even greater extent. This species is also invariably used in the food industry as a spice and as a flavouring additive to alcoholic beverages and teas. A novelty is the possibility of using extracts from various parts of the plant in the production of cosmetics. The species is approved by the European CosIng database to be used for this purpose. In the light of contemporary research, *A. abrotanum* appears to be an attractive species with valuable medicinal, culinary, and cosmetic qualities.

## Figures and Tables

**Table 1:** Chemical composition of A. abrotanum essential oil.

Group of Compounds	Compounds	Plant’s Part	Origin	Extraction Method	Extraction Yield	Concentration	Refs.
**Sesquiterpenoids**	β-caryophyllene	aerial parts	Japan	*n*-hexane	nd *	3.37% (Fukuoka), 1.35% (Okinawa)	[44]
		leaves	Cuba	hd **	nd	0.7%	[45]
		aerial parts	Egypt	hd	2.2–10.1 mL/ 100 g	0.5–2.8%	[46]
		nd	Germany	nd	nd	0.7%	[47]
	β-elemene	leaves	Cuba	hd	nd	0.1%	[44]
		aerial parts	Japan	*n*-hexane	nd	0.08% (Fukuoka), nd (Okinawa)	[45]
		aerial parts	Egypt	hd	2.2–10.1 mL/ 100 g	1.7–2.4%	[46]
	germacrene D	leaves	Cuba	hd	1.9%	0.1%	[44]
		aerial parts	Japan	*n*-hexane	nd	12.47% (Fukuoka), 25.05% (Okinawa)	[45]
		aerial parts	Egypt	hd	2.2–10.1 mL/ 100 g	1.8–2.7%	[46]
		nd	Germany	nd	nd	3.4%	[47]
		aerial parts	Iraq	*n*-hexane	nd	0.27%	[48]
		nd,	Crimea	nd	nd	0.68–1.43%	[49]
	7-epi-silphiperfol-5-ene	aerial parts	Japan	*n*-hexane	nd	2.24% (Fukuoka), 0.5% (Okinawa)	[45]
	silphiperfol-4,7 (14)-diene	aerial parts	Japan	*n*-hexane	nd	nd (Fukuoka), trace (Okinawa)	[45]
		nd	Germany	nd	nd	0.3%	[47]
	silphiperfol-5-ene	aerial parts	Japan	*n*-hexane	nd	1.09% (Fukuoka), 0.82% (Okinawa)	[45]
		nd	Germany	nd	nd	0.7%	[47]
	silphiperfol-5-en-3-ol A	aerial parts	Japan	*n*-hexane	nd	1.65% (Fukuoka), 5.79% (Okinawa)	[45]
	silphiperfol-5-en-3-one A	aerial parts	Japan	*n*-hexane	nd	68.94% (Fukuoka), 56.11% (Okinawa)	[45]
		nd	Germany	nd	nd	18.9%	[47]
	silphiperfol-5-en-3-one B	aerial parts	Japan	*n*-hexane	nd	5.37% (Fukuoka), 5.03% (Okinawa);	[45]
		nd	Germany	nd	nd	2.7%	[47]
	silphiperfol-6-α-ol	aerial parts	Japan	*n*-hexane	nd	1.40% (Fukuoka), 1.84%(Okinawa)	[45]
	silphiperfolen isomer	nd	Germany	nd	nd	0.7%	[47]
	β-copaene	aerial parts	Japan	*n*-hexane	nd	0.09% (Fukuoka), 0.22% (Okinawa)	[45]
	δ-elemen	aerial parts	Japan	*n*-hexane	nd	1.22% (Fukuoka), 1.81% (Okinawa)	[45]
	(*E*)-nerolidol	leaves	Cuba	hd	1.9%	<0.1%	[44]
	(*E*)-β-damascenone	leaves	Cuba	hd	1.9%	0.1%	[44]
	α-dehydro-ar-himachalene	leaves	Cuba	hd	1.9%	2.5%	[44]
	γ-dehydro-ar-himachalene	leaves	Cuba	hd	1.9%	0.4%	[44]
	δ-cadinene	leaves	Cuba	hd	1.9%	0.1%	[44]
	humulene epoxide I	leaves	Cuba	hd	1.9%	<0.1%	[44]
	T-muurolol	aerial parts	Egypt	hd	2.2–10.1 mL/ 100 g	0.4–1.6%	[46]
	α-cadinol	aerial parts	Egypt	hd	2.2–10.1 mL/ 100 g	0.7–1.8%	[46]
	cedrene	aerial parts	Iraq	*n*-hexane	nd	1.38%	[48]
	3-carene	aerial parts	Iraq	*n*-hexane	nd	2.00%	[48]
	citronellol	aerial parts	Iraq	*n*-hexane	nd	0.38%	[48]
	caryophyllene	aerial parts	Iraq	*n*-hexane	nd	0.54%	[48]
		nd	Crimea	nd	nd	0.15%	[49]
	farnesyl butanoate	aerial parts	Lithuania	hd	nd	0.32–0.38%	[9]
	α-cubebene	aerial parts	Lithuania	hd	nd	0.08–0.34%	[9]
	(*E*)-β-farnesene	aerial parts	Lithuania	hd	nd	0.15–0.19%	[9]
	bisabolone	aerial parts	Lithuania	hd	nd	0.17–0.46%	[9]
	β-cubebene	aerial parts	Lithuania	hd	nd	0.11–0.12%	[9]
	α-epi-7-epi-5-eudesmol	aerial parts	Lithuania	hd	nd	0.00–0.34%	[9]
	γ-eudesmol acetate	aerial parts	Lithuania	hd	nd	0.80–2.18%	[9]
	isospathulenol	aerial parts	Lithuania	hd	nd	0.26–0.80%	[9]
	isogermacrene D	aerial parts	Lithuania	hd	nd	5.23–15.67%	[9]
	germacren-D-4-ol	aerial parts	Lithuania	hd	nd	0.00–0.44%	[9]
	epi-longipinanol	aerial parts	Lithuania	hd	nd	0.00–0.92%	[9]
	davanone B	aerial parts	Lithuania	hd	nd	0.00–0.67%	[9]
	δ-amorphene	aerial parts	Lithuania	hd	nd	0.00–17.85%	[9]
	β-selinene	aerial parts	Lithuania	hd	nd	0.00–0.09%	[9]
		leaves	Cuba	hd	1.9%	0.3%	[44]
	α-humulene	aerial parts	Lithuania	hd	nd	0.32–0.51%	[9]
		leaves	Cuba	hd	1.9%	0.1%	[44]
		aerial parts	Japan	*n*-hexane	nd	1.25% (Fukuoka), 0.04% (Okinawa)	[45]
		nd	Germany	nd	nd	1.8%	[47]
	bicyclogermacrene	aerial parts	Lithuania	hd	nd	0.27–0.83%	[9]
		leaves	Cuba	hd	1.9%	<0.1%	[44]
		aerial parts	Japan	*n*-hexane	nd	0.78% (Fukuoka), 1.58% (Okinawa)	[45]
		nd	Crimea	nd	nd	0.23–0.30%	[49]
	caryophyllene oxide	aerial parts	Lithuania	hd	nd	0.45–1.15%	[9]
		leaves	Cuba	hd	1.9%	0.8%;	[44]
		aerial parts	Iraq	n-hexane	nd	1.99%	[48]
		nd	Crimea	nd	nd	0.11–0.19%	[49]
	α-copaene	aerial parts	Lithuania	hd	nd	0.09–0.13%;	[9]
		aerial parts	Japan	n-hexane	nd	0.05% (Fukuoka), 0.16% (Okinawa)	[45]
		aerial parts	Egypt	hd	2.2–10.1 mL/100 g	0.6–2.9%	[46]
	β-bourbonene	aerial parts	Lithuania	hd	nd	0.00–0.52%	[9]
		nd	Crimea	nd	nd	0.24%	[49]
	davanone	aerial parts	Egypt	hd	2.2–10.1 mL/100 g	1.4–2.1%	[46]
	aromadendrene	aerial parts	Lithuania	hd	nd	2.39–7.44%	[9]
	*cis*-davanone	aerial parts	Romania	hd	nd	5.2%	[47]
		aerial parts	Romania	dichloromethane followed by hd	nd	7.4%	[47]
	davanon ether	aerial parts	Romania	hd	nd	0.9%	[47]
	davana ether	aerial parts	Romania	hd	nd	3.2%	[47]
	artedouglasia oxide A	aerial parts	Romania	hd	nd	2.20%	[47]
		aerial parts	Romania	dichloromethane followed by hd	nd	1.80%	[47]
	artedouglasia oxide B	aerial parts	Romania	hd	nd	1.3%;	[47]
		aerial parts	Romania	dichloromethane followed by hd	nd	1.0%	[47]
	artedouglasia oxide D	aerial parts	Romania	hd	nd	1.0%	[47]
		aerial parts	Romania	dichloromethane followed by hd	nd	0.6%	[47]
	nordavanone	aerial parts	Romania	hd	nd	3.0%	[47]
		aerial parts	Romania	dichloromethane followed by hd	nd	5.4%	[47]
	artedouglasia C	aerial parts	Lithuania	hd	nd	0.20–0.23%	[9] [47]
		aerial parts	Romania	hd	nd	1.7%	
		aerial parts	Romania	dichloromethane followed by hd	nd	1.1%	
	eudesma-5-en-11-ol	nd	Crimea	nd	nd	0.14–0.37%	[49]
	α-eudesmol	nd	Crimea	nd	nd	0.19–4.85%	[49]
	*trans*-α-bisabolen	nd	Crimea	nd	nd	1.09%	[49]
	cadinol					0.72–1.19%	[49]
	guaiol	nd	Crimea	nd	nd	0.19–2.40%	[49]
	nerolidol	nd	Crimea	nd	nd	0.28%	[49]
	spathulenol	nd	Crimea	nd	nd	0.17–0.21%	[49]
	α-bisabolol	aerial parts	Egypt	hd	2.2–10.1 mL/ 100 g	0.5–1.7%	[46]
		nd	Crimea	nd	nd	3.52–4.64%	[49]
	β-eudesmol	aerial parts	Egypt	hd	2.2–10.1 mL/ 100 g	12.4–12.9%	[46]
		nd	Crimea	nd	nd	0.12–0.26%	[49]
**Monoterpenoids**	chrysanthenone	leaves	Cuba	hd	1.9%	2.7%	[44]
	α-thujene	leaves	Cuba	hd	1.9%	0.5%	[44]
	*p*-cymenene	leaves	Cuba	hd	1.9%	<0.1%	[44]
	neryl propionate	leaves	Cuba	hd	1.9%	0.2%	[44]
	*trans*-sabinol	leaves	Cuba	hd	1.9%	5.1%	[44]
	isobornyl propionate	leaves	Cuba	hd	1.9%	0.1%	[44]
	α-thujone	leaves	Cuba	hd	1.9%	<0.1%	[44]
	*E*-β-ocimene	leaves	Cuba	hd	1.9%	0.3%	[44]
	*Z*-β-ocimene	leaves	Cuba	hd	1.9%	<0.1%	[44]
	cuminyl acetate	leaves	Cuba	hd	1.9%	<0.1%	[44]
	*cis*-carvyl acetate	leaves	Cuba	hd	1.9%	0.1%	[44]
	bornyl acetate	leaves	Cuba	hd	1.9%	1.6%	[44]
	neryl isobutanoate	leaves	Cuba	hd	1.9%	0.4%	[44]
	isobornyl formate	nd	Germany	nd	nd	0.1%	[47]
	sabinene	leaves	Cuba	hd	1.9%	4.4%	[44]
		aerial parts	Egypt	hd	2.2–10.1 mL/ 100 g	0.8–1.3%	[46]
		nd	Germany	nd	nd	0.1%	[47]
		aerial parts	Iraq	n-hexane	nd	0.77%	[48]
		nd	Crimea	nd	nd	0.34–3.58%	[49]
	myrcene	leaves	Cuba	hd	1.9%	0.9%	[44]
		aerial parts	Egypt	hd	2.2–10.1 mL/ 100 g	2.1–2.7%	[46]
		nd	Crimea	nd	nd	0.33%	[49]
	*p*-cymene	leaves	Cuba	hd	1.9%	<0.1%	[44]
		aerial parts	Egypt	hd	2.2–10.1 mL/ 100 g	0.7–1.5%	[46]
		nd	Germany	nd	nd	7.8%	[47]
		nd	Crimea	nd	nd	0.39–1.69%	[49]
	α-terpineol	leaves	Cuba	hd	1.9%	8.2%	[44]
		aerial parts	Egypt	hd	2.2–10.1 mL/ 100 g	1.3–1.9%	[46]
		nd	Crimea	nd	nd	0.22–1.43%	[49]
		nd	Germany	nd	nd	0.2%	[47]
	γ-terpinene	leaves	Cuba	hd	1.9%	2.6%	[44]
		nd	Crimea	nd	nd	0.73–1.06%	[49]
		aerial parts	Egypt	hd	2.2–10.1 mL/ 100 g	1.5–2.9%	[46]
	4-terpineol	leaves	Cuba	hd	1.9%	5.9%	[44]
		aerial parts	Egypt	hd	2.2–10.1 mL/ 100 g	0.8–0.9%	[46]
		nd	Crimea	nd	nd	2.27–3.72%	[49]
		nd	Germany	nd	nd	1.8%	[47]
	eugenol	leaves	Cuba	hd	1.9%	0.2%	[44]
		aerial parts	Egypt	hd	2.2–10.1 mL/ 100 g	1.6–2.1%	[46]
		nd	Crimea	nd	nd	0.16%	[49]
	limonene	leaves	Cuba	hd	1.9%	<0.1%	[44]
		aerial parts	Egypt	hd	2.2–10.1 mL/ 100 g	1.6–2.3%	[46]
		nd	Crimea	nd	nd	0.16–0.48%	[49]
	geranyl isobutanoate	leaves	Cuba	hd	1.9%	<0.1%	[44]
		nd	Crimea	nd	nd	0.17–0.79%	[49]
	*trans*-carvyl acetate	leaves	Cuba	hd	1.9%	0.1%	[44]
		nd	Crimea	nd	nd	0.12–0.49%	[49]
	terpinolene	leaves	Cuba	hd	1.9%	0.9%	[44]
		nd	Crimea	nd	nd	0.21–0.26%	[49]
	*cis*-sabinene hydrate	aerial parts	Lithuania	hd	nd	0.04–0.11%	[9]
		leaves	Cuba	hd	1.9%	0.3%	[44]
		nd	Crimea	nd	nd	0.14–0.97%	[49]
		nd	Germany	nd	nd	0.3%	[47]
	myrtenol	aerial parts	Lithuania	hd	nd	0.0–0.9%	[9]
		aerial parts	Iraq	*n*-hexane	nd	0.47%	[48]
		nd	Crimea	nd	nd	0.09–0.39%	[49]
	*trans*-ocimene	nd	Crimea	nd	nd	0.13–0.29%	[49]
	lavandulyl butanoate	aerial parts	Lithuania	hd	nd	0.83–4.70%	[9]
	3-thujanol	aerial parts	Lithuania	hd	nd	0.00–0.21%	[9]
	β-myrcene	aerial parts	Lithuania	hd	nd	0.05–0.11%	[9]
	β-ocimene	aerial parts	Lithuania	hd	nd	0.00–0.07%	[9]
	*trans*-β-ocimene	aerial parts	Lithuania	hd	nd	0.00–0.05%	[9]
	1,4-cineole	aerial parts	Lithuania	hd	nd	4.12–13.14%	[9]
	β-phellandrene	aerial parts	Lithuania	hd	nd	0.35–0.44%	[9]
	*trans*-ocimenol	aerial parts	Lithuania	hd	nd	0.00–0.08%	[9]
	α-terpinolene	aerial parts	Lithuania	hd	nd	0.07–0.09%	[9]
	*cis*-chrysanthenol	aerial parts	Lithuania	hd	nd	0.49–0.84%	[9]
	*trans*-pinocamphone	aerial parts	Lithuania	hd	nd	0.00–0.11%	[9]
	lavandulol	aerial parts	Lithuania	hd	nd	0.00–0.30%	[9]
	4-tujanol	aerial parts	Lithuania	hd	nd	0.52–1.00%	[9]
	*cis*-piperitol	aerial parts	Lithuania	hd	nd	0.00–0.17%	[9]
	*cis*-chrysanthenyl acetate	aerial parts	Lithuania	hd	nd	0.15–0.21%	[9]
	δ-terpineol acetate	aerial parts	Lithuania	hd	nd	0.10–0.14%	[9]
	α-terpinyl acetate	aerial parts	Lithuania	hd	nd	0.05–0.16%	[9]
	β-myrcene	aerial parts	Lithuania	hd	nd	0.00-0.05%	[9]
	*E*-myrtenol	aerial parts	Lithuania	hd	nd	0.00–0.9%	[9]
	lavandulyl caproate	aerial parts	Lithuania	hd	nd	0.28–1.21%	[9]
	lavandulyl isovalerate	aerial parts	Lithuania	hd	nd	0.33–0.65%	[9]
	*trans*-β-ocimene	aerial parts	Lithuania	hd	nd	0.00–0.05%	[9]
	*trans*-chrysanthenyl acetate	aerial parts	Germany	nd	nd	1.0%	[47]
	*trans*-sabinyl acetate	aerial parts	Lithuania	hd	nd	0.29–0.50%	[9]
		leaves	Cuba	hd	1.9%	33.4%	[44]
	α-phellandrene	leaves	Cuba	hd	1.9%	0.1%	[44]
		aerial parts	Lithuania	hd	nd	0.05–0.25%	[9]
	camphene	aerial parts	Lithuania	hd	nd	0.10–0.64%	[9]
		leaves	Cuba	hd	1.9%	1.7%	[44]
		nd	Crimea	nd	nd	2.42–7.20%	[49]
		nd	Germany	nd	nd	2.7%	[47]
	α-pinene	aerial parts	Lithuania	hd	nd	0.09–0.20%	[9]
		leaves	Cuba	hd	1.9%	1.1%	[44]
		aerial parts	Egypt	hd	2.2–10.1 mL/ 100 g	0.7–1.9%	[46]
		nd	Crimea	nd	nd	1.1–1.8%	[49]
		nd	Germany	nd	nd	0.1%	[47]
	α-terpinene	leaves	Cuba	hd	1.9%	2.4%	[44]
		aerial parts	Lithuania	hd	nd	0.21–0.31%	[9]
		nd	Crimea	nd	nd	0.27–0.50%	[49]
		nd	Germany	nd	nd	0.9%	[47]
	*trans*-sabinene hydrate	nd	Crimea	nd	nd	0.24–1.15%	[49]
		nd	Germany	nd	nd	0.3%	[47]
	linalool	aerial parts	Lithuania	hd	nd	0.08–0.11%	[9]
		nd	Crimea	nd	nd	0.2%	[49]
	1,8-cineole	leaves	Cuba	hd	1.9%	4.3%	[44]
		aerial parts	Lithuania	hd	nd	0.0–13.0%	[9]
		aerial parts	Iraq	n-hexane extraction	nd	10.05%	[48]
		nd	Crimea	nd	nd	15.46–33.19%	[49]
		nd	Germany	nd	nd	24.5%	[47]
	2-hydroxy-1,8-cineole	aerial parts	Egypt	hd	2.2–10.1 mL/ 100 g	37.9–38.7%	[46]
	borneol	leaves	Cuba	hd	1.9%	1.6%	[44]
		aerial parts	Egypt	hd	2.2–10.1 mL/ 100 g	0.5–2.8%;	[46]
		aerial parts	Iraq	*n*-hexane	nd	8.96%	[48]
		nd	Crimea	nd	nd	2.16–3.29%	[49]
		nd	Germany	nd	nd	9.3%	[47]
	camphor	aerial parts	Lithuania	hd	nd	0.86–1.33%	[9]
		aerial parts	Iraq	*n*-hexane	nd	6.31%	[48]
		aerial parts	Egypt	hd	2.2–10.1 mL/ 100 g	11.8–12.1%	[46]
		nd	Crimea	nd	nd	20.33–44.63%	[49]
		nd	Germany	nd	nd	3.5%	[47]
	1-terpineol	aerial parts	Romania	hd	nd	1.6%	[47]
	*cis*-β-terpineol	aerial parts	Romania	hd	nd	1.6%	[47]
		aerial parts	Romania	dichloromethane followed by hd	nd	0.8%	[47]
	*trans*-piperitol	aerial parts	Iraq	*n*-hexane	nd	0.38%	[48]
		aerial parts	Romania	hd	nd	1.2%	[47]
	piperitone	aerial parts	Lithuania	hd	nd	20.38–38.48%	[9]
		aerial parts	Romania	hd	nd	0.5%	[47]
	terpenyl acetate	aerial parts	Egypt	hd	2.2–10.1 mL/ 100 g	0.7–2.2%	[46]
	myrtanal	aerial parts	Egypt	hd	2.2–10.1 mL/ 100 g	0.4–1.1%	[46]
	α-terpenyl acetate	nd	Crimea	nd	nd	0.13–0.31%	[49]
	verbenyl acetate	nd	Crimea	nd	nd	0.20–0.44%	[49]
	*trans*-carveol	nd	Crimea	nd	nd	0.23–0.33%	[49]
	myrtenal	nd	Crimea	nd	nd	0.08–0.12%	[49]
		nd	Germany	nd	nd	0.37%	[47]
	3 (10) -carene-2-ol	nd	Crimea	nd	nd	1.10–1.19%	[49]
	*α*-thujenal	nd	Crimea	nd	nd	0.26%	[49]
	2 (10) -pinen-2-one	nd	Crimea	nd	nd	3.75%	[49]
	*cis*-carvone	nd	Crimea	nd	nd	0.13%	[49]
	ment-1,5-dien-7-ol	nd	Crimea	nd	nd	0.54%	[49]
	pinocarvone	nd	Crimea	nd	nd	1.26%	[49]
	*p*-menth-1-en-8-ol	nd	Crimea	nd	nd	2.16%	[49]
	verbenol	nd	Crimea	nd	nd	2.83–3.22%	[49]
	sabinaketone	nd	Crimea	nd	nd	0.24%	[49]
	*p*-menth-2-en-1-ol	nd	Crimea	nd	nd	0.16–0.32%	[49]
	cembrene	aerial parts	Lithuania	hd	nd	0.11–0.12%	[9]
	tricyclene	nd	Crimea	nd	nd	0.09–0.2%	[49]
		nd	Germany	nd	nd	0.08%	[47]
	*β*-pinene	nd	Crimea	nd	nd	0.26–0.97%	[49]
		nd	Germany	nd	nd	0.3%	[47]
**Diterpenoids**	phytol isomer	aerial parts	Romania	hd	nd	1.2%	[47]
	lupeol	aerial parts	Iraq	*n*-hexane	nd	7.0%	[48]
**Triterpenoids**	agarospirol	nd	Crimea	nd	nd	0.19–2.40%	[49]
**Spiroterpenoids**	methyleugenol	leaves	Cuba	hd	1.9%	<0.1%	[44]
		nd	Crimea	nd	nd	0.30–1.58%	[49]
**Phenylpropanoid derivatives**	estragol (methyl chavicol)	leaves	Cuba	hd	1.9%	<0.1%	[44]
		aerial parts	Romania	hd	nd	0.9%	[47]
		aerial parts	Romania	dichloromethane followed by hd	nd	0.8%	[24]
	elemicine	nd	Crimea	nd	nd	0.12–0.26%	[49]
**Jasmonates**	methyl *cis*-jasmonate	aerial parts	Egypt	hd	2.2–10.1 mL/ 100 g	1.9–2.6%	[46]
**Other compounds**	1-octen-3-ol	aerial parts	Lithuania	hd	nd	0.16–0.18%	[9]
		nd	Crimea	nd	nd	0.43–0.73%	[49]
	(*Z*)-jasmone,	aerial parts	Lithuania	hd	nd	0.15–0.18%	[9]
	nonanal	aerial parts	Lithuania	hd	nd	0.00–0.33%	[9]
	1,4-dimethyl-4-propyl-2-one-1-(2)–cyclo-hexene	nd	Crimea	nd	nd	0.39–0.54%	[49]
	2,2,3-trimethyl-3-cyclopentene-1-acetaldehyde	nd	Crimea	nd	nd	0.20–0.23%	[49]
	*cis*-jasmone	nd	Crimea	nd	nd	0.28–0.45%	[49]
	*cis*-arbusculone	aerial parts	Lithuania	hd	nd	0.35–2.15%	[9]
		aerial parts	Romania	hd	nd	0.7%	[47]
	(*E*)-2-hexenal	leaves	Cuba	hd	1.9%	0.4%	[44]
	(*Z*)-3-hexenol	leaves	Cuba	hd	1.9%	<0.1%	[44]
	2-phenylacetaldehyde	leaves	Cuba	hd	1.9%	<0.1%	[44]
	hexanal	leaves	Cuba	hd	1.9%	<0.1%	[44]
	heptanal	leaves	Cuba	hd	1.9%	<0.1%	[44]
	α-(*E*)-ionone	leaves	Cuba	hd	1.9%	<0.1%	[44]
	methyl *p*-anisate	leaves	Cuba	hd	1.9%	0.1%	[44]
	isobutanoate ester of anisic acid	leaves	Cuba	hd	1.9%	<0.1%	[44]
	isopergol	aerial parts	Iraq	*n*-hexane	nd	1.14%	[48]
	4-methylpent-2-enolide	aerial parts	Romania	hd	nd	15.7%	[47]
		aerial parts	Romania	dichloromethane followed by hd	nd	1.7%	[47]
	lavender lactone	aerial parts	Romania	hd	nd	2.6%	[47]
	*trans*-arbusculone	aerial parts	Romania	hd	nd	0.6%	[47]

* nd—no data; **—hd—hydrodistillation.

**Table 2:** Chemical composition of *A. abrotanum*.

Group of Compounds	Compounds	References
Sesquiterpene lactones	santonin	[50]
	artemisinin	[33,37,40]
**Flavonoids**	rutoside	[33]
	apigenin, artemisetin, hyperoside, isoquercitrin, kaempferol, quercetol, luteolin, myricetin, patuletin	[40]
	quercetin	[40,55]
	centaureidine, casticin	[56]
**Coumarines**	isofraxidine	[33,37]
	umbelliferone	[33,37,50]
	scopoletin	[33,37,50,56]
	herniarin	[37]
	esculetin	[37,50]
	coumarin	[50]
**Phenolic acids**	ferulic acid, gentisic acid, caftaric acid, *p*-coumaric acid, sinapic acid	[40]
	chlorogenic acid	[33,37,40,50,55]
	isochlorogenic acid, protocatechuic acid, rosmarinic acid, syryngic acid, vanillic acid	[55]
	caffeic acid	[33,37,40,50,55]
**Sterols**	24β-ethylcholesta-6 (7), 20 (21)-dien-3β-ol	[52]
**Resins**	nd *	[33]
**Tannins**	nd	[33,37,54]
**Alkaloids**	abrotine	[33,37,57]
**Other compounds**	*cis*-jasmone	[50]

* nd—no data.

**Table 3:** Directions of biological activity of *A. abrotanum* confirmed by scientific research.

Activity	Mechanism of Action	References
antibacterial and antifungal	Lethal effect of *A. abrotanum* ethanolic extract on the bacteria *Bacillus stearothermophilus*, *Micrococcus luteus*, *Klebsiella pneumoniae*, *Pseudomonas cepacia*, *Salmonella typhi*, and the fungi *Candida albicans*, *Saccharomyces cerevisiae*, *Trichosporon beigelii*.	[65]
	Lethal effect of the essential oil of *A. abrotanum* herb on *Candida albicans*.	[47]
	Inhibition of the growth of *Staphylococcus aureus*, *Escherichia coli* and *Candida albicans* by components of *A. abrotanum* essential oil, incl. borneol, cymene, camphor, terpineol, 1,8-cineole, and aromadendrene.	[63]
	Inhibition of the growth of *Escherichia coli*, *Pseudomonas aeruginosa*, *Staphylococcus aureus*, *Proteus vulgaris* bacteria by the essential oil of the herb *A. abrotanum.* Some activity against *Aspergillus flavus.*	[66]
	Inhibition of the growth of the bacteria: *Listeria monocytogenes*, *Staphylococcus aureus*, *Escherichia coli*, *Bacillus cereus*, *Pseudomonas aeruginosa*, *Micrococcus flavus*, and the fungi *Penicillium ochrochloron*, *Penicillium funiculosum*, *Candida albicans*, *Aspergillus ochraceus*, *Aspergillus niger*, and *Aspergillus flavus.*	[55]
	Moderate inhibition of the growth of bacteria: *Streptococcus pyogenes*, *Streptococcus agalactiae*, *Streptococcus gordonii*, *Enterococcus faecalis*, *Escherichia coli*, *Citrobacter freundii*, *Pseudomonas aeruginosa*; methicilin suseptible: *Staphylococcus aureus and Staphylococcus epidermis*; methicilin resistant: *Staphylococcus aureus* and *Staphylococcus haemolyticus* and macrolides resistant: *Propionibacterium acnes* strains under influence of *A. abrotanum* herb ethanolic extract. Decrement of *Candida albicans*, *Candida tropicalis* colonies and *Aspergillus niger spore germination.* Synergistic action of *A. abrotanum* herb ethanolic extract with erythromycin against *Staphylococcus aureus* with efflux mechanism of MLS-resistance.	[12]
antioxidative	Moderate antioxidant activity of *A. abrotanum* ethanolic extract in the test with DPPH.	[40]
	Reducing potential and inhibition of lipid peroxidation by the essential oil from the herb of *A. abrotanum*.	[66]
	Reducing potential of methanolic extract from *A. abrotanum* herb, in particular its components, rosmarinic acid, isochlorogenic acid and quercitrin.	[55]
antitumour	Decrease in the survival of neoplastic cells of the RD (rhabdomyosarcoma) line by the components of *A. abrotanum* essential oil such as borneol, cymene, camphor, terpineol, 1,8-cineole and aromadendrene.	[63]
	Methanolic extract of *A. abrotanum* leaves and its components (incl. chlorogenic acid and isochlorogenic acid) inhibit the proliferation of cells of the Jurkat line (T-lymphoblastic leukemia line), MCF-7 line (breast adenocarcinoma line), Hela line (cervical adenocarcinoma line), HT-29 line (colorectal adenocarcinoma line).	[55]
alleviating allergy symptoms	Relief of symptoms of allergic rhinitis with possible concomitant allergic conjunctivitis, relief of symptoms of bronchial obstruction and symptoms of exercise-induced asthma by using a nasal spray with a mixture of essential oils and flavonoids present in *Artemisia abrotanum*.	[56]
insect repellent	Toluene extract from the herb *A. abrotanum* and the individual components of the extract show an insect repellent effect against *Ixodes ricinus* and *Aedes aegypti*.	[50]
against animal parasites	Reduction in the number of eggs of *Hymenolepis nana* (dwarf tapeworm), *Syphacia obvelata* and *Aspiculuris tetraptera* (rodent pinworms) in the faeces of mice after administration of ethanolic extract from *A. abrotanum* leaves.	[67]
antiplasmodial	Notable antiprotozoal activity against *P. falciparum* under influence of *A. abrotanum*-AgNPs	[68]
